# A case report of malignant hypertension and multiorgan dysfunction during immunotherapy for gallbladder cancer

**DOI:** 10.3389/fonc.2025.1658621

**Published:** 2025-10-13

**Authors:** Caroline Anthon, Hugo Pierret, Frederic Houssiau, Selda Aydin, Astrid De Cuyper, Cédric Van Marcke, Marc Van Den Eynde, Filomena Mazzeo, Frank Cornelis, Rachel Galot, Francois P. Duhoux, Jean-François Baurain, Emmanuel Seront

**Affiliations:** 1 Institut Roi Albert II, Medical Oncology, Cliniques Universitaires Saint-Luc, Brussels, Belgium; 2 Department of Rhumatology, Cliniques Universitaires Saint-Luc, Brussels, Belgium; 3 Department of Pathology, Cliniques Universitaires Saint-Luc, Brussels, Belgium

**Keywords:** immune checkpoint inhibitor, thrombotic microangiopathy, scleroderma renalcrisis, immune-related adverse event, systemic sclerosis

## Abstract

We report the case of a 77-year-old woman with metastatic gallbladder cancer who initially received adjuvant capecitabine following surgery. During this period, she developed a facial rash, associated with a positive antinuclear antibody (ANA) with a titer of 1:320. Six months later, disease recurrence prompted treatment with gemcitabine, cisplatin, and durvalumab. Shortly after completing six cycles of chemo-immunotherapy, she presented with rapidly progressive dyspnea, severe hypertension, thrombotic microangiopathy (TMA; confirmed on renal pathology), and multiorgan dysfunction, including hepatic and muscular involvement. Laboratory workup revealed a strongly positive ANA titer (1:1280) corresponding to strongly positive anti-Th/To antibodies. Although the clinical presentation was highly suggestive of scleroderma renal crisis (SRC), it remained challenging to determine whether this was an immune-related adverse event, a paraneoplastic manifestation, or an exacerbation of a pre-existing autoimmune condition. This case illustrates the diagnostic and therapeutic complexity of autoimmune phenomena in oncology and highlights the importance of thorough autoimmune screening and multidisciplinary collaboration before and during immunotherapy.

## Highlights

- Immune checkpoint inhibitors can unmask or trigger systemic autoimmune diseases, including systemic sclerosis, even without cutaneous features.
- Scleroderma renal crisis may present with malignant hypertension and thrombotic microangiopathy; early diagnosis is crucial.
- Anti-Th/To antibodies, though rare, are highly specific for systemic sclerosis and may guide diagnosis in atypical presentations.
- High-dose corticosteroids can worsen renal outcomes in scleroderma renal crisis; ACE inhibitors are the mainstay of treatment.
- Baseline autoimmune screening and close monitoring during immunotherapy are essential, especially in patients with prior autoimmune signs.

## Introduction

Gallbladder cancer (GBC) is a rare malignancy in Europe, with an incidence of approximately 1.6 cases per 100,000. It predominantly affects women and often presents with non-specific symptoms, contributing to delayed diagnosis, poor prognosis, and low five-year survival rates (1). The treatment landscape for advanced biliary tract cancers has evolved with the introduction of immune checkpoint inhibitors (ICIs), increasingly used in combination with platinum-based chemotherapy (2). While ICIs offer clinical benefit, they can also trigger a wide range of immune-related adverse events (irAEs)—from organ-specific toxicities (e.g., thyroiditis, colitis, hepatitis) to more complex systemic autoimmune syndromes (3).

Multisystem irAEs involving the vascular, renal, hepatic, and muscular systems pose a significant diagnostic challenge in patients receiving combination therapy. These complications may result from overlapping mechanisms such as immune activation, chemotherapy toxicity, paraneoplastic syndromes, or reactivation of latent autoimmunity. The scarcity of data on connective tissue disease-like irAEs further complicates clinical decision-making. We report a case of metastatic GBC treated with chemo-immunotherapy, complicated by multiorgan dysfunction and autoimmune seroconversion. This case underscores the diagnostic complexity of systemic irAEs and highlights the importance of multidisciplinary evaluation in cancer patients receiving ICIs (3).

## Case description

**Figure 1:**
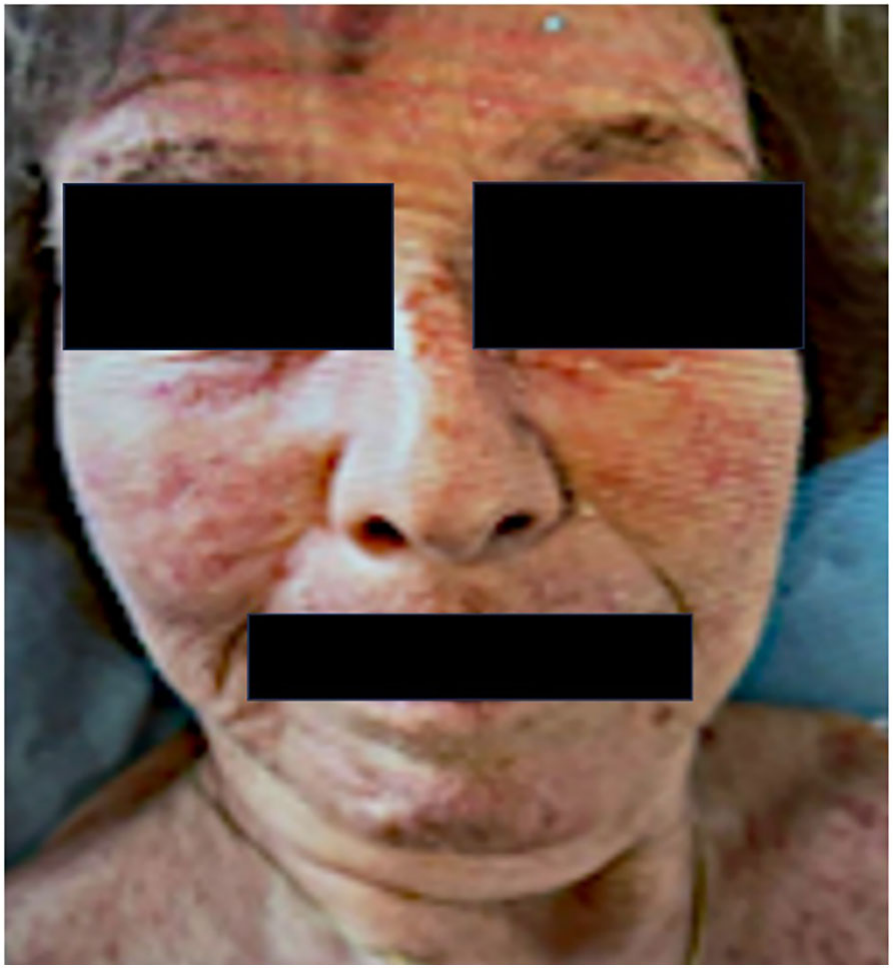
Photosensitive rash involving the face, occurring during chemotherapy.

A 77-year-old woman underwent cholecystectomy for acute cholecystitis. Histopathology revealed gallbladder adenocarcinoma (pT2aN1M0), prompting hepatic bi-segmentectomy and lymphadenectomy. She received adjuvant capecitabine, discontinued after four cycles due to coronary vasospasm and gastrointestinal toxicity. During treatment, she developed a photosensitive rash with low-titer antinuclear antibodies (ANA, 1:320), possibly due to cutaneous lupus (**Figure 1**). The rash resolved spontaneously, and in the absence of systemic features, no further workup was performed. Daily blood pressure was at this time within upper normal range, not exceeding 140mmHg.

Six months later, in April 2024, imaging revealed retroperitoneal metastatic recurrence. The patient was started on gemcitabine, cisplatin, and durvalumab. Pre-treatment laboratory tests showed normal blood counts and renal function. ANA was moderately positive (1:160), without clinical signs of autoimmunity. She completed six cycles, during which she developed progressive fatigue and anemia. At the end of chemotherapy, hemoglobin had dropped to 7.5 g/dL with 1.3% schistocytes. Platelets and renal function remained normal and tensional profiles did not exceed in consultation 150mmHg systolic. As a chemotherapy-related hemogram toxicity, including a low-grade gemcitabine-induced microangiopathy, chemotherapy was discontinued, and durvalumab was continued alone with a close follow-up.

## Timeline

**Figure 2:**
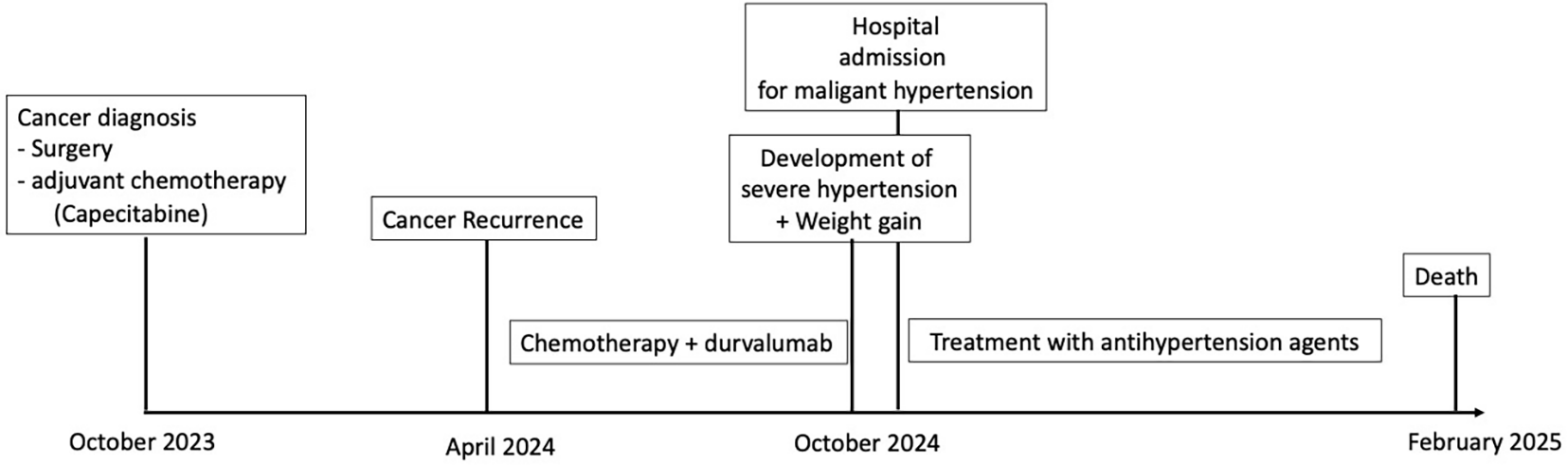
Timeline.

Two weeks later—before the next scheduled durvalumab dose in October 2024—the patient presented to the emergency department with rapidly worsening dyspnea, diffuse edema, and a 15-kg weight gain over 10 days. Her blood pressure was 210/110 mmHg. Examination revealed bilateral basal hypoventilation, lower limb edema, and mild *de novo* acrocyanosis. No cutaneous lesions were noted on the face. She also reported recent arthralgia and myalgia without muscle weakness; no acrosyndrome was reported. Timeline is represented in **Figure 2**.

## Diagnosis

**Figure 3:**
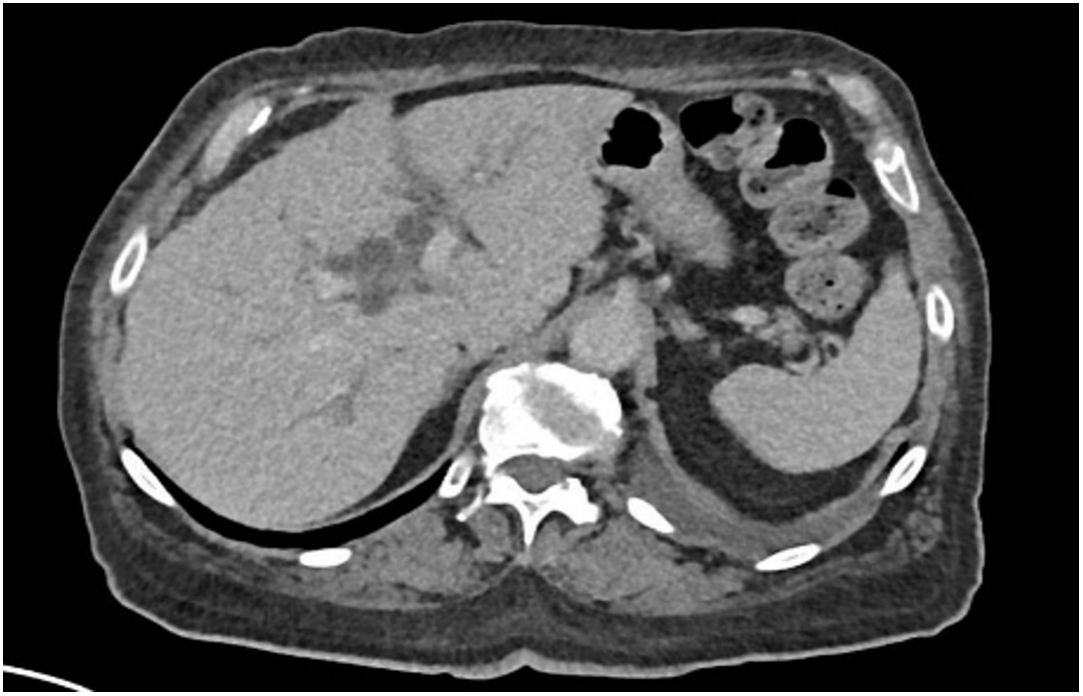
Moderate pleural effusions and mild intrahepatic bile duct dilatation, without clear evidence of progression on Computed Tomography Scan.

Laboratory investigations showed hemoglobin of 5.8 g/dL, 3.5% schistocytes, undetectable haptoglobin, and severe thrombocytopenia (20,000/mm³), consistent with thrombotic microangiopathy (TMA). Liver enzymes were elevated (Alanine transaminase 302 U/L, Aspartate transaminase 300 U/L, alkaline phosphatase 378 U/L, GGT 302 U/L), as well as Creatine phospho-kinases (1,259 U/L) and troponin-T (145 U/L). Creatinine had risen from 0.8 to 1.6 mg/dL. CT scan showed moderate bilateral pleural effusions and mild intrahepatic bile duct dilatation, without clear evidence of progression (**Figure 3**). Fundoscopy revealed grade 3 hypertensive retinopathy. Cardiac MRI and echocardiography showed preserved function and no signs of myocarditis.

High-dose corticosteroids (2 mg/kg/day), ACE inhibitors, and loop diuretics were initiated, based on a potential immune-related hepatitis, myositis, nephritis and TMA. A calcium channel blocker and beta-blocker were added after four days due to persistent hypertension. By day five, while still on high dose of steroids, CPK levels decreased by more than 50%, but anemia, thrombocytopenia, and hemolysis persisted in the same range. Progressive elevation in bilirubin and liver enzymes prompted endoscopic retrograde cholangiography, which revealed tumor-related biliary compression. Biliary stenting was performed, leading to normalization of liver enzymes by day nine.

**Figure 4:**
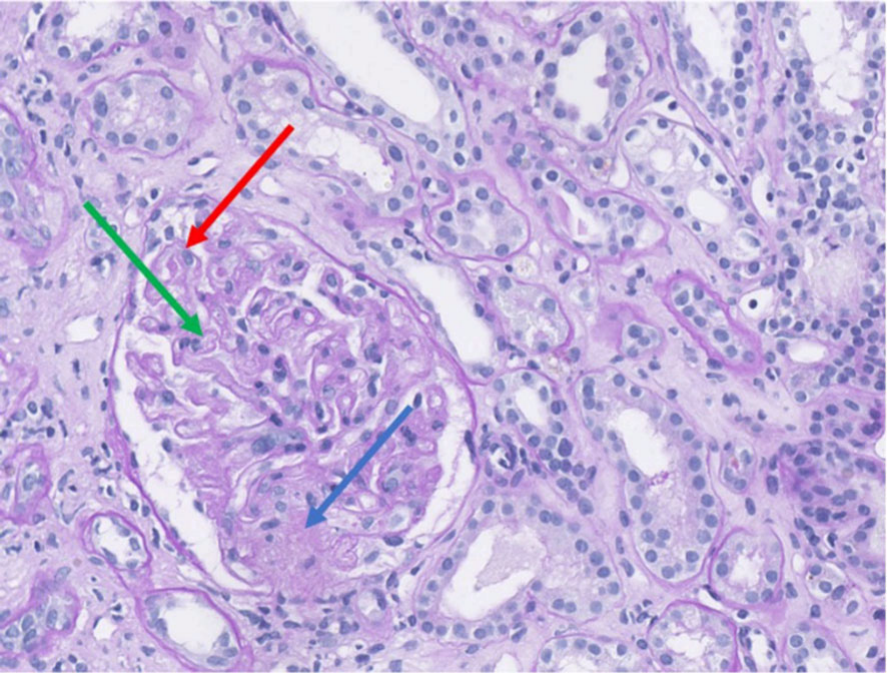
Renal biopsy demonstrating histological features of thrombotic microangiopathy (Periodic Acid–Schiff staining, magnification ×35). The glomeruli exhibit duplication of the glomerular basement membrane (red arrow) associated with endothelial swelling. Segmental ischemic lesion (green arrow) and mesangiolysis (blue arrow) are consistent with acute thrombotic microangiopathy.

Despite improvement in hepatic and muscular parameters, renal function declined between days 7 and 15. ANA titer were increased to 1:1280, and anti-Th/To antibodies were found to be strongly positive (100/100). Renal biopsy confirmed TMA. These findings were consistent with a scleroderma renal crisis (SRC) (**Figure 4**).

Given the suspected SRC, the confirmed tumor progression, and stabilization of liver and muscle enzymes, corticosteroids were tapered rapidly. Blood pressure remained elevated but stable on a combination of antihypertensives and diuretics. The patient experienced modest clinical improvement, including weight loss and improved respiratory function, although renal function continued to decline.

A second-line FOLFOX regimen was considered in light of disease progression but could not be initiated due to intercurrent complications, including influenza infection requiring oxygen therapy, a fall resulting in arm fracture, and overall functional decline. With no viable treatment options remaining, she was transitioned to palliative care and died three months later from progressive metastatic disease and functional deterioration. Till the end, tensional profiles remained high (>150mmHg despite ACE inhibitors, calcium channel blocker and beta-blocker).

## Discussion

This case illustrates the diagnostic complexity of systemic manifestations in cancer patients receiving ICIs. Six months after initiating chemo-immunotherapy for metastatic gallbladder cancer, our patient developed malignant hypertension, acrosyndrome, multiorgan dysfunction (hemolysis, hepatic, renal, and muscular involvement), and a marked autoimmune response, including antinuclear antibodies.

The initial presentation raised several differential diagnoses. Gemcitabine-induced TMA was considered, as the patient had shown progressive anemia and schizocytes during the final chemotherapy cycles. However, the delayed onset and clinical worsening three weeks after discontinuation, coupled with evolving multiorgan dysfunction, were atypical for a self-limited drug-induced TMA. A paraneoplastic TMA was also conceivable in metastatic gallbladder cancer, yet there was no unequivocal radiologic progression at TMA onset, biliary decompression improved cholestasis but not malignant hypertension or renal decline, and the serologic profile better aligns with systemic sclerosis.

The emergence of additional features—acrosyndrome, elevated ANA, raised hepatic and muscular enzymes, and renal deterioration—strengthened the suspicion of an evolving systemic autoimmune condition. Latent autoimmunity unmasked by intercurrent illness could explain the earlier photosensitive rash with low-titer ANA. The subsequent identification of anti-Th/To antibodies anchored the diagnosis within the systemic-sclerosis spectrum: anti-Th/To are uncommon overall (~3–6% of SSc) and are enriched in limited cutaneous SSc, often with minimal skin thickening and a propensity for pulmonary involvement and renal complication, including SRC (4). Importantly, this represents an atypical serologic profile for SRC and we therefore interpret anti-Th/To positivity as supportive of an underlying SSc diathesis rather than a direct or common association with SRC in this case.

SSc is a chronic autoimmune disorder characterized by immune dysregulation, vascular dysfunction, and progressive fibrosis. Its pathogenesis involves endothelial injury that triggers immune-fibroblast cross-talk, promoting chronic inflammation and tissue remodeling (5). ANA positivity occurs in over 90% of patients and may precede clinical manifestations. Whether this situation represented *de novo* ICI-induced SSc (ICI-SSc), or an unmasking of a preclinical autoimmune condition, or a paraneoplastic phenomenon remains uncertain. ICI-related SSc is rare; a recent literature review described four cases occurring after a median of 7 to 14 months of exposure to pembrolizumab or nivolumab, often with atypical presentations and rapid progression (6). In our patient, durvalumab had been started six months before symptom onset, placing her well within this expected timeframe.

A pre-existing autoimmune predisposition, exacerbated by durvalumab, is also plausible. The patient had previously developed a lupus-like rash and low-titer ANA (1:320) during capecitabine treatment. Supporting this, a large post-marketing retrospective analysis reported a 24% flare rate in patients with pre-existing SSc treated with ICIs, including a case of new-onset SRC despite the absence of corticosteroid use (7, 8).

This case also underscores the management challenges of multisystem irAEs during ICI therapy. High-dose corticosteroids were initially prescribed under the assumption of immune-related hepatitis or myositis. At that stage, the SSc diagnosis had not yet been established, and the patient presented with malignant hypertension. In retrospect, earlier recognition of SSc might have led to a more cautious approach, prioritizing ACE inhibitors for the SRC and delaying or minimizing corticosteroids.

Corticosteroids are first-line for most moderate–severe irAEs. By contrast, when SRC is suspected, high-dose glucocorticoids have been associated with precipitating/worsening SRC, and management should prioritize immediate ACE-inhibitor therapy with rapid up-titration (typically captopril); any steroids should be minimized and reserved only for compelling overlapping irAEs, with prompt taper (9). Whether this risk extends to ICI-SSc requires further investigation.

While the temporal relationship to PD-L1 blockade and the clinical phenotype support an immune-checkpoint–associated mechanism, causality cannot be established in a single case. We therefore frame this as probable ICI-associated SSc/SRC, acknowledging that gemcitabine-TMA and paraneoplastic TMA remain credible alternative explanations and may have contributed to the overall picture (10, 11).

Unfortunately, our patient died rapidly, without any significant improvement in the anemia, thrombopenia, creatinine and hemolysis. Due to cancer-unrelated complications, her general status deteriorated, and no other systemic treatment was given.

## Patient perspective

The patient expressed surprise and concern upon learning that the immunotherapy used to treat her cancer could lead to serious complications such as kidney injury and severe hypertension. The diagnosis of a possible autoimmune condition affecting the kidneys was unexpected and distressing. Despite the need to discontinue cancer treatment, she expressed appreciation for the multidisciplinary care she received and the improvement in her condition. She hoped that sharing her experience could help raise awareness of such rare complications and support earlier diagnosis and management in other patients.

## Conclusion

This case represents a probable instance of ICI-induced systemic sclerosis, presenting initially as scleroderma renal crisis. The combination of malignant hypertension, TMA, multiorgan involvement, anti-Th/To positivity, and acrosyndrome builds a compelling argument for this diagnosis. While alternative etiologies—such as gemcitabine toxicity, paraneoplastic syndrome, or latent autoimmunity—cannot be entirely excluded, the temporal association with durvalumab, immune serology, and partial response to corticosteroids all support an immune-mediated mechanism. Clinicians should be aware that SRC can be an initial presentation of ICI-SSc, even in the absence of classic skin findings. ACE inhibitors remain the mainstay of SRC management, while decisions about continuing immunotherapy must balance oncologic benefit against the risk of autoimmune exacerbation.

## Data Availability

The original contributions presented in the study are included in the article/supplementary material. Further inquiries can be directed to the corresponding author.
