# Correction: A case report of malignant hypertension and multiorgan dysfunction during immunotherapy for gallbladder cancer

**DOI:** 10.3389/fonc.2026.1771057

**Published:** 2026-01-22

**Authors:** Caroline Anthon, Hugo Pierret, Frederic Houssiau, Selda Aydin, Astrid De Cuyper, Cédric Van Marcke, Marc Van Den Eynde, Filomena Mazzeo, Frank Cornelis, Rachel Galot, Francois P. Duhoux, Jean-François Baurain, Emmanuel Seront

**Affiliations:** 1 Institut Roi Albert II, Medical Oncology, Cliniques Universitaires Saint-Luc, Brussels, Belgium; 2 Department of Rhumatology, Cliniques Universitaires Saint-Luc, Brussels, Belgium; 3 Department of Pathology, Cliniques Universitaires Saint-Luc, Brussels, Belgium

**Keywords:** immune checkpoint inhibitor, thrombotic microangiopathy, scleroderma renalcrisis, immune-related adverse event, systemic sclerosis

Author Selda Aydin was omitted as an author in the published article. The correct author list reads:

“Caroline Anthon^1,†^, Hugo Pierret^1,†^, Frederic Houssiau^2^, Selda Aydin^3,^ Astrid De Cuyper^1^, Cédric Van Marcke^1^, Marc Van Den Eynde^1^, Filomena Mazzeo^1^, Frank Cornelis^1^, Rachel Galot^1^, Francois P Duhoux^1^, Jean-François Baurain^1^, Emmanuel Seront ^1,*^”

Affiliation 3 “Department of Pathology, Cliniques Universitaires Saint-Luc, Brussels, Belgium” was omitted for author Selda Aydin. This affiliation has now been added for author Selda Aydin.

The Author Contributions Statement has been corrected to read: “CA: Writing – review & editing, Writing – original draft. HP: Writing – review & editing, Writing – original draft. FH: Writing – original draft, Writing – review & editing. SA: Writing – original draft, Writing – review & editing. AD: Writing – review & editing, Writing – original draft. CV: Writing – review & editing, Writing – original draft. MV: Writing – review & editing, Writing – original draft. FM: Writing – review & editing, Writing – original draft. FC: Writing – review & editing, Writing – original draft. RG: Writing – original draft, Writing – review & editing. FD: Writing – review & editing, Writing – original draft. J-FB: Writing – review & editing, Writing – original draft. ES: Resources, Investigation, Software, Conceptualization, Funding acquisition, Supervision, Writing – review & editing, Data curation, Project administration, Formal Analysis, Writing – original draft, Validation, Methodology, Visualization”.

There was a mistake in **Figure 4** as published. There was two parts in the figure as published. The corrected **Figure 4** appears below.

There was a mistake in the caption of **Figure 4** as published. The caption has some errors in the published version. The corrected caption of **Figure 4** appears below.

**Figure 4:**
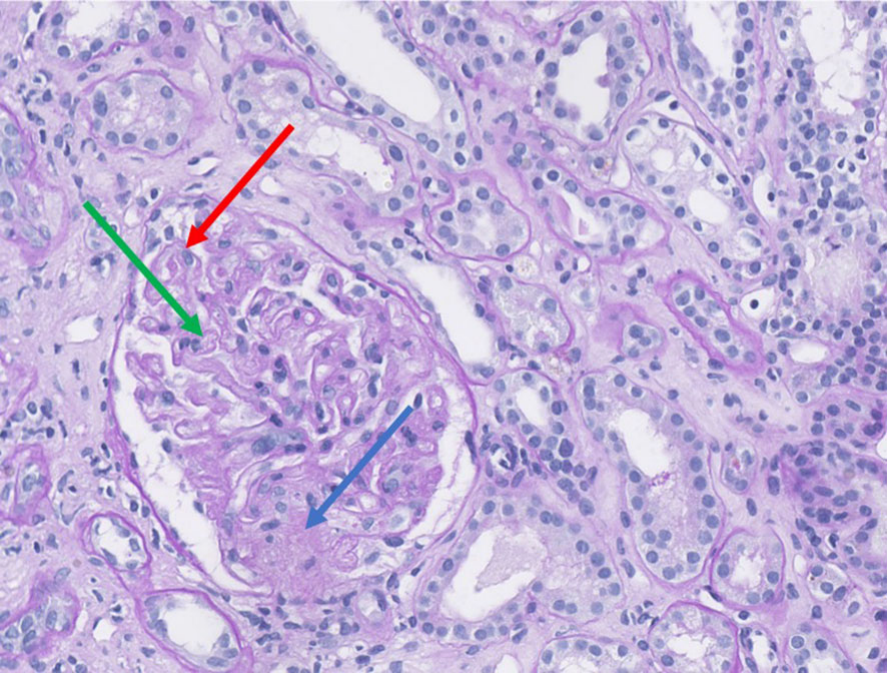
Renal biopsy demonstrating histological features of thrombotic microangiopathy (Periodic Acid–Schiff staining, magnification ×35). The glomeruli exhibit duplication of the glomerular basement membrane (red arrow) associated with endothelial swelling. Segmental ischemic lesion (green arrow) and mesangiolysis (blue arrow) are consistent with acute thrombotic microangiopathy.

“Renal biopsy demonstrating histological features of thrombotic microangiopathy (Periodic Acid–Schiff staining, magnification ×35). The glomeruli exhibit duplication of the glomerular basement membrane (red arrow) associated with endothelial swelling. Segmental ischemic lesion (green arrow) and mesangiolysis (blue arrow) are consistent with acute thrombotic microangiopathy”

The original version of this article has been updated.

